# Prediagnostic plasma concentrations of organochlorines and risk of B-cell non-Hodgkin lymphoma in envirogenomarkers: a nested case-control study

**DOI:** 10.1186/s12940-017-0214-8

**Published:** 2017-02-16

**Authors:** Rachel S. Kelly, Hannu Kiviranta, Ingvar A. Bergdahl, Domenico Palli, Ann-Sofie Johansson, Maria Botsivali, Paolo Vineis, Roel Vermeulen, Soterios A. Kyrtopoulos, Marc Chadeau-Hyam, on behalf of the EnviroGenoMarkers project consortium, Hector C. Keun, Toby J. Athersuch, Karin vanVeldhoven, Bo Jonsson, Beatrice Melin, Per Lenner, Panagiotis Georgiadis, Christina Papadopoulou, Aristotelis Chatziioannou, Ioannis Valavanis, Carlotta Sacerdote, Lützen Portengen, Fatemeh Saberi-Hosnijeh, Dennie G. A. J. Hebels, Jos C. S. Kleinjans, Theo M. C. M. de Kok, Ralph Gottschalk, Danitsja van Leeuwen, Leen Timmermans, Göran Hallmans, Benedetta Bendinelli, Vittorio Krogh, Rosario Tumino, Salvatore Panico, Manolis Kogevinas, Euripides G. Stephanou, Antonis Myridakis, Lucia Fazzo, Marco De Santis, Pietro Comba, Panu Rantakokko, Riikka Airaksinen, Päivi Ruokojärvi, Mark Gilthorpe, Sarah Fleming, Thomas Fleming, Yu-Kang Tu, Wei J. Chen, Wen-Chung Lee, Chuhsing Kate Hsiao, Kuo-Liong Chien, Po-Hsiu Kuo, Hung Hung, Shu-Fen Liao, Thoma Lundh

**Affiliations:** 1 Channing Division of Network Medicine, Department of Medicine, Brigham and Women’s Hospital and Harvard Medical School, 181 Longwood Avenue, Boston, MA 02115 USA; 2 Department of Epidemiology and Biostatistics, Imperial College London, London, UK; 3 National Institute for Health and Welfare Chemicals and Health Unit, Neulanen Research Centre, Neulaniementie 4, FI-70210 Kuopio, Finland; 4 Occupational and Environmental Medicine, Department of Public Health and Clinical Medicine, Umeå University, Umeå, Sweden; 5 Molecular and Nutritional Epidemiology Unit, Cancer Prevention and Research Institute (ISPO), Florence, Italy; 6 Department of Radiation Sciences, Oncology, Umeå University, Umeå, Sweden; 7 National Hellenic Research Foundation, Institute of Biology, Pharmaceutical Chemistry and Biotechnology, Athens, Greece; 8 HuGeF Foundation, Turin, Italy; 9 Institute for Risk Assessment Sciences, Division of Environmental Epidemiology, Utrecht University, Utrecht, The Netherlands

**Keywords:** Non Hodgkin lymphoma, Polychlorinated biphenyls, DDE, HCB, Organochlorines, EnviroGenoMarkers

## Abstract

**Background:**

Evidence suggests a largely environmental component to non-Hodgkin’s lymphoma (NHL). Persistent organic pollutants (POPs) including polychlorinated biphenyls (PCBs), DDE and HCB have been repeatedly implicated, but the literature is inconsistent and a causal relationship remains to be determined.

**Methods:**

The EnviroGenoMarkers study is nested within two prospective cohorts EPIC-Italy and the Northern Sweden Health and Disease Study. Six PCB congeners, DDE and HCB were measured in blood plasma samples provided at recruitment using gas-chromatography mass spectrometry. During 16 years follow-up 270 incident cases of B-cell NHL (including 76 cases of multiple myeloma) were diagnosed. Cases were matched to 270 healthy controls by centre, age, gender and date of blood collection. Cases were categorised into ordered quartiles of exposure for each POP based on the distribution of exposure in the control population. Logistic regression was applied to assess the association with risk, multivariate and stratified analyses were performed to identify confounders or effect modifiers.

**Results:**

The exposures displayed a strong degree of correlation, particularly amongst those PCBs with similar degrees of chlorination. There was no significant difference (*p* < 0.05) in median exposure levels between cases and controls for any of the investigated exposures. However under a multivariate model PCB138, PCB153, HCB and DDE displayed significant inverse trends (Wald test *p*-value <0.05). Under stratified analyses these were determined to be driven by males and by the Diffuse Large B-Cell Lymphoma subtype. When considering those in the highest levels of exposure (>90^th^ percentile) the association was null for all POPs

**Conclusion:**

We report no evidence that a higher body burden of PCBs, DDE or HCB increased the risk of subsequent NHL diagnosis. Significantly inverse associations were noted for males with a number of the investigated POPs. We hypothesize these unexpected relationships may relate to the subtype composition of our population, effect modification by BMI or other unmeasured confounding. This study provides no additional support for the previously observed role of PCBs, DDE and HCB as risk factors for NHL.

**Electronic supplementary material:**

The online version of this article (doi:10.1186/s12940-017-0214-8) contains supplementary material, which is available to authorized users.

## Background

The non-Hodgkin's lymphomas (NHL) are a heterogeneous collection of lymphoproliferative B or T-cell malignancies that typically present as a solid tumour of lymphoid cells in the glands [1]. Currently more than 50 different subtypes of B and T-cell NHL have been defined, differing in both clinic-pathological and biological characteristics [2]. Yet despite their widespread prevalence and high mortality rate, relatively little is known about the aetiology of NHL [3].

Immune dysfunction is the underlying basis of lymphomagenesis and the most well established risk factor to date is immunosuppression [4–6]. However immunosuppressed patients in fact account for only a small fraction of cases, and the epidemiological evidence suggests a largely environmental component to the aetiology of NHL [7]. The reported increase in incidence of NHL, and particularly of the more highly aggressive subtypes, in the UK and most other Western countries since the 1980s had been noted to mirror the worldwide usage trends of a number of suspected chemical risk factors, including polychlorinated biphenyls (PCBs). A plateauing of this increase was observed to occur following the banning of many of these substances in much of the world [8]. Similarly, an excess of cases has been reported in those who are occupationally or residentially exposed to the suggested environmental risk factors [9]. However, the literature is inconsistent and to date, few definitive environmental risk factors for NHL have been agreed upon

The EnviroGenoMarkers project [10] is a nested case-control study within two cohorts with prospectively collected blood samples. The study aimed at the development and application of a new generation of biomarkers to aid the study of the role of environmental agents in disease. It utilises the large-scale application of multiple –omics technologies to validate novel predictive disease biomarkers and to explore the association of these biomarkers with environmental pollutants using the “*meet-in-the-middle*” approach [11]. “*meet-in-the-middle*” employs parallel analysis of exposure-biomarker relationships and metabolite-biomarker relationships, in an attempt to understand the causal pathway. The first step of this approach is to determine the exposure–disease relationship. The aim of this present study was to determine the association between NHL and prediagnositic blood plasma concentrations of ten environmental pollutants including six PCB congeners, DDT, DDE, HCB and BDE-47.

## Methods

### Study subjects

The EnviroGenoMarkers study is based on 540 participants from two existing prospective cohort studies: EPIC-Italy (*n* = 168) [12] and the Northern Sweden Health and Disease Study (NSHDS, *n* = 372) [13] which have been described in detail previously. This study was approved by the committee on research ethics at the relevant institutions in accordance with the Declaration of Helsinki of the World Medical Association. All participants signed an informed consent form agreeing to provide detailed information on their dietary and life-style habits at recruitment and to provide blood samples for use in future research.

Both cohorts recruited healthy volunteers who underwent standardised protocols including detailed questionnaires, anthropometric measurements, medical record review and collection of blood samples. Recruitment for EPIC-Italy took place between 1993 and 1998, and for the NSHDS between 1990 and 2006. In EPIC-Italy standardized procedures were used to identify newly diagnosed cases of cancer based on automated linkages to cancer and mortality registries, municipal population offices and hospital discharge systems, with the exception of Naples where follow up information was collected through periodic personal contact. In the NSHDS invasive cancers were identified by linkage with the Swedish Cancer Registry and the local Northern Sweden Cancer Registry.

For each lymphoma case that occurred within up to 16 years follow up a suitable cancer-free control was selected by incidence matching on sex, age (+/− 2.5 years), centre and date of blood extraction (+/− 6 months) from the remaining cancer-free populations. More than 95% of participants also had the same fasting status as their matched pair at blood extraction. Information from the two studies was integrated into a single database and calibrated. Lymphoma cases were classified into subtypes according to the SEER ICD-0-3 morphology codes [14].

### Exposure assessment

Exposure to persistent organic pollutants (POPs) was assessed using blood plasma. Blood samples were collected in citrate (EPIC-Italy) or heparin (NSHDS) tubes and processed by centrifugation on the day of collection (EPIC-Italy) or within one hour of collection (NSHDS). Aliquots (0.5 ml) were stored in liquid nitrogen tanks at −196 °C in Italy and −80 °C in NSHDS.

All samples were measured at the National Institute for Health and Welfare Chemicals and Health Unit, Neulanen Research Centre, Finland The quantification of POPs was performed by an Agilent 6890 gas chromatograph (GC) connected to a Waters Autospec Ultima high resolution mass spectrometer (HRMS). The GC column used was DB-5MS (J&W Scientific, 30 m, ID 0.25 mm, 0.25 μm), and the splitless mode was used for injection. For 43 EPIC-Italy matched pairs a slightly different procedure was used involving an Agilent 7000B gas chromatography triple quadruple mass spectrometry (GC-MS/MS) system, with a DB-5MS UI (J&W Scientific, 20 m, ID 0.18 mm, 0.18 μm) GC column and the solvent vent mode. For quality control purposes in each batch of samples (43 batches with HRMS, 20 batches with MS/MS) two reagent blanks were additionally prepared and the average result of the blank samples subtracted from the results of the real samples. Furthermore two control samples of Standard Reference Material 1589a (PCBs, Pesticides, BDEs, Dioxins/Furans in Humans) from the National Institute of Standards and Technology, were also included in each batch. Unadjusted volume-based concentrations of POPs, which have been found to correlate well with lipid based concentrations, are presented [15].

### Statistical analysis

All analyses were conducted on the total population as well as stratified by cohort (EPIC-Italy and NSHDS) and by sex where appropriate. The differences in baseline characteristics between cases and controls were determined using Student’s *t*-test and chi-squared test for continuous outcomes and categorical outcomes respectively.

Exposure concentrations were explored using non-parametric methods due to their typically non-Gaussian distributions. Differences in concentrations between the cohorts and by sex specific analyses were assessed using the ranksum test while the differences between cases and controls were assessed using the Wilcoxon signed rank test to take matching into account. Correlation between exposure levels was assessed using Pearson’s correlation coefficient.

Cases were categorised into ordered quartiles based on the distribution of exposure in the control population. A further binary variable was created to assess whether blood concentration exceeding ‘highly exposed’ levels, defined as being above the 90th percentile of the control exposure distribution, was associated with greater risk by comparing it to those in the lowest quartile. For the remaining analyses, stratified by subtype, age group, BMI category and time to diagnosis, exposure levels were log transformed to normalise the distributions.

In the basic model, conditional logistic regression analyses were used to calculate odds ratios and 95% confidence intervals accounting for the matching factors. Tests for trend were performed using the Wald test statistic based on the continuous quartiles. Where all controls were included to maximise power, unconditional logistic regression adjusting for the matching variables was used. Confounding was assessed by additionally running an adjusted model; potential confounders were identified as BMI (kg/m^2^), height (cm), educational level (as a proxy for socioeconomic status), vegetable intake (g/day), dairy intake (g/day), protein intake (g/day), total fat intake (g/day) and alcohol consumption (g/day), based on both a significant (*p* < 0.05) association with cumulative POP concentration in the total population and a reported association with NHL in the literature [16, 17] to ensure biological plausibility for the included confounders.

All analyses were performed using Stata 11.3, the level of statistical significance was set at 0.05 and all tests were two tailed.

## Results

A total of 281 incident cases were diagnosed during follow-up; 84 from EPIC Italy and 197 from NSHDS. Only cases of B-cell NHL were included in the final analyses, resulting in the exclusion of six cases of Hodgkin’s Lymphoma, one case of T-cell NHL, four cases with unknown subtype and their matched controls (*n* = 11 case-control pairs). There were no significant differences between cases and controls for any of the baseline variables investigated (Table 1). The median time from blood collection to diagnosis was 6.6 years in NSHDS and 5.0 years in Epic-Italy, There was no association between time to NHL diagnosis and body burden of any of the pollutants amongst the cases (*p*-value > 0.05).

**Table 1:** Baseline characteristics of cases and controls

Baseline variable		Case (*n* = 270) [n(%)]	Control (*n* = 270) [n(%)]	Difference (*p*-value)
Cohort	EPIC-Italy	84 (31.1)	84 (31.1)	
	NSHDS	186 (68.9)	186 (68.9)	
Sex	Male	133 (49.3)	133 (49.3)	
	Female	137 (50.7)	137 (50.7)	
Mean Age	(yrs)	53.08	53.09	0.989
Mean Height	(cm)	169.55	168.24	0.120
Mean Weight	(kg)	76.48	75.09	0.267
BMI	Underweight	1 (0.4)	0 (0.0)	
	Normal	104 (38.5)	109 (40.4)	
	Overweight	121 (44.8)	118 (43.7)	
	Obese	40 (14.8)	39 (14.4)	
	unknown	4 (1.5)	4 (1.5)	0.883
Smoking status	Never	121 (44.8)	134 (49.6)	
	Former	90 (33.3)	72 (26.7)	
	Current	57 (21.1)	54 (20)	
	unknown	2 (0.7)	10 (3.7)	0.269
Highest educational level	None	4 (1.5)	1 (0.4)	
	Primary	94 (34.8)	98 (36.3)	
	Technical/professional	68 (25.2)	54 (20.0)	
	Secondary	52 (19.3)	64 (23.7)	
	University/college	47 (17.4)	42 (15.6)	
	unknown	5 (1.9)	11 (4.1)	0.202
Cambridge physical activity index	Inactive	80 (29.6)	75 (27.8)	
	Moderately inactive	106 (39.3)	95 (35.2)	
	Moderately active	69 (25.6)	76 (28.2)	
	Active	14 (5.2)	23 (8.5)	
	unknown	1 (0.4)	1 (0.4)	0.510

Chi2 tests excluded the unknown category

The mean, median and range of exposure concentrations in cases and controls for each investigated pollutant are shown in Table 2. There was no significant difference (*p* < 0.05) in median exposure levels between cases and controls according to the Wilcoxon signed rank test for any of the investigated exposures (Table 2). The investigated exposures displayed a strong degree of correlation (Additional file 1: Table S1), particularly amongst those PCBs with similar degrees of chlorination.

**Table 2:** Mean, median and range of exposure concentrations of six PCB congeners, HCB, DDT, DDE and BDE-47

Exposure	LOQ	Cases (*n* = 270)				% Controls (*n* = 270)^a^				Difference *p*-value^b^
		% < LOQ	Median	Mean	Range	% < LOQ	Median	Mean	Range	
PCB118	5 pg/ml	0.0%	134.7	182	(8.5, 901.6)	0.0%	147.7	175.7	(11.6, 832.1)	0.991
PCB138	5 pg/ml	0.0%	530.0	605.6	(11.0, 1810.1)	0.0%	562.3	636.4	(51.2, 2675.4)	0.276
PCB153	4 pg/ml	0.0%	1007.8	1150.9	(32.5, 3308.0)	0.0%	1086	1205.5	(120.6, 4334.1)	0.239
PCB156	2 pg/ml	0.0%	90.6	102	(19.8, 307.8)	0.0%	95.6	106.7	(15.5, 394.9)	0.186
PCB170	4 pg/ml	0.0%	326.4	375.2	(62.7, 1082.3)	0.0%	357.6	389.4	(50.4, 1291.2)	0.284
PCB180	3 pg/ml	0.0%	696.6	788.8	(139.7, 2708.8)	0.0%	727.8	807.9	(100.1, 2431.5)	0.284
HCB	25 pg/ml	0.0%	257.8	398.1	(62.5, 3604.0)	0.0%	289.2	432.5	(64.5, 3882.2)	0.198
DDT	200 pg/ml	85.9%	261.8	331.7	(206.6, 825.8)	86.7%	298.2	399.8	(218.8, 1636.9)	0.865
DDE	5 pg/ml	0.0%	2478.7	4038.4	(16.4, 30992.8)	0.0%	2675.3	4200.1	(76.4, 23858.3)	0.374
BDE-47	15 pg/ml	84.1%	23.5	34.2	(15.1, 133.4)	81.1%	25.8	95.1	(15.3, 2063.3)	0.657

*LOQ* limit of quantification

^a^PCB analyses were based on 269 controls as one participant with exposure levels above the 99^th^ percentile was excluded

^b^Differences between median concentrations in cases and controls according to the Wilcoxon signed-rank test for paired samples

For BDE-47 and DDT more than 80% of the population had levels that could not be quantified and therefore these exposures were excluded from further analyses. There was no significant difference in the proportion of cases and controls above and below the LOQ for either BDE-47 (*p* = 0.706) or DDT (*p* = 0.425). One participant, a male control from the NSHDS was excluded from the PCB analyses as an outlier as his exposure levels were far above the 99th percentile of the distribution for all congeners.

Table 3 shows the association between increasing quartiles of exposure and risk of NHL, both under the basic conditional model, and when adjusting for potential confounders. Although under the basic model a number of significant inverse associations were observed there was no evidence of a dose-response trend. When adjusting on confounders, PCB138, PCB153, HCB and DDE displayed inverse trends.

**Table 3:** Association between NHL and quartiles of measured body burden of six PCB congeners, specified PCB functional groups, HCB and DDE

Exposure	Quartile	n. Ca	n. Co	OR	95% CI	*P*-value	p for trend	OR(adj)	95% CI	*P*-value	p for trend
PCB118	Q1 (8.46, 94.05)	80	68	1				1			
	Q2 (94.25, 148.04)	68	67	0.76	(0.44,1.32)	0.332		0.57	(0.31,1.08)	0.084	
	Q3 (148.37, 219.85)	42	66	0.45	(0.24,0.84)	0.012^*^		0.35	(0.17,0.72)	0.004^*^	
	Q4 (219.91, 901.63)	80	67	0.96	(0.49,1.87)	0.909	0.571	0.67	(0.3,1.49)	0.332	0.168
PCB138	Q1 (10.97, 396.03)	86	67	1				1			
	Q2 (399.73, 562.09)	60	67	0.67	(0.41,1.08)	0.102		0.53	(0.31,0.91)	0.021^*^	
	Q3 (562.64, 778.15)	63	67	0.63	(0.37,1.1)	0.104		0.50	(0.27,0.94)	0.031^*^	
	Q4 (778.44, 2675.35)	61	67	0.60	(0.33,1.08)	0.089	0.093	0.38	(0.18,0.78)	0.009^*^	0.010^*^
PCB153	Q1 (32.47, 812.10)	88	68	1				1			
	Q2 (813.60, 1083.97)	59	66	0.60	(0.36,1.03)	0.064		0.56	(0.31,1.02)	0.058	
	Q3 (1088.49, 1489.97)	60	67	0.54	(0.3,0.98)	0.044^*^		0.47	(0.24,0.93)	0.031^*^	
	Q4 (1493.70, 4334.12)	63	67	0.55	(0.3,1.04)	0.066	0.078	0.37	(0.17,0.78)	0.009^*^	0.011^*^
PCB156	Q1 (15.50, 69.69.62)	75	67	1				1			
	Q2 (69.95, 95.44)	71	67	0.88	(0.49,1.56)	0.654		0.9	(0.48,1.71)	0.754	
	Q3 (95.76, 126.68)	51	67	0.59	(0.31,1.11)	0.100		0.56	(0.27,1.16)	0.118	
	Q4 (126.87, 394.89)	73	67	0.86	(0.45,1.64)	0.644	0.545	0.69	(0.33,1.45)	0.326	0.228
PCB170	Q1 (50.39, 257.50)	73	67	1				1			
	Q2 (257.62, 358.81)	82	68	0.99	(0.59,1.66)	0.967		0.9	(0.51,1.6)	0.718	
	Q3 (359.10, 468.52)	43	66	0.48	(0.25,0.92)	0.028^*^		0.37	(0.17,0.79)	0.010^*^	
	Q4 (468.55, 1291.23)	72	67	0.83	(0.45,1.53)	0.55	0.356	0.59	(0.28,1.23)	0.160	0.091
PCB180	Q1 (100.08, 533.51)	72	67	1				1			
	Q2 (533.84, 730.03)	78	68	1.03	(0.61,1.73)	0.926		1	(0.55,1.81)	0.999	
	Q3 (730.81, 966.02)	46	66	0.57	(0.31,1.05)	0.071		0.45	(0.21,0.95)	0.035^*^	
	Q4 (966.55, 2708,83)	74	67	0.94	(0.52,1.69)	0.827	0.595	0.68	(0.32,1.42)	0.300	0.168
HCB	Q1 (62.47, 181.87)	85	67	1				1			
	Q2 (184.78, 288.81)	62	67	0.58	(0.32,1.03)	0.064		0.52	(0.27,1.02)	0.057	
	Q3 (289.49, 474.86)	59	67	0.47	(0.24,0.94)	0.032^*^		0.28	(0.12,0.65)	0.003^*^	
	Q4 (477.00, 3882.19)	64	66	0.49	(0.22,1.07)	0.074	0.061	0.35	(0.14,0.87)	0.023^*^	0.014^*^
DDE	Q1 (16.40, 1308.26)	81	67	1				1			
	Q2 (1310.23, 2666.60)	62	67	0.65	(0.37,1.14)	0.137		0.55	(0.28,1.05)	0.07	
	Q3 (2675.27, 5523.58)	68	68	0.67	(0.37,1.21)	0.186		0.40	(0.20,0.84)	0.015^*^	
	Q4 (5528.29, 30992.76)	59	66	0.55	(0.28,1.08)	0.082	0.106	0.34	(0.15,0.77)	0.010^*^	0.008^*^

OR – conditional logistic regression accounting for matching factors

OR^(adj)^ – conditional logistic regression additionally adjusting for BMI, height, educational level, vegetables, dairy, protein, total fat, alcohol

^*^Significant at the 95% confidence level

Stratified analyses by gender (Table 4) indicated that these inverse associations were heavily influenced by males who displayed multiple dose-dependent inverse trends with the investigated POPs. This was not observed in females. In analyses stratified by cohort (Additional file 1: Table S2) inverse associations with these POPs were restricted to the Swedish cohort.

**Table 4:** Association between log-transformed body burden of six PCB congeners, specified PCB functional groups, HCB and DDE, levels and NHL risk stratified by sex

POP by quartile	Males									Females								
	Ca/Co	OR	95% CI	*P*-value	p for trend	OR^(adj)^	95% CI	*P*-value	p for trend	Ca/Co	OR	95% CI	*P*-value	p for trend	OR^(adj)^	95% CI	*P*-value	p for trend
PCB118	47/34	1				1				31/34	1				1			
	35/32	0.6	(0.27,1.32)	0.202		0.69	(0.28,1.72)	0.424		35/34	1.28	(0.59,2.81)	0.534		1.09	(0.46,2.6)	0.846	
	22/33	0.32	(0.13,0.78)	0.013*		0.21	(0.07,0.65)	0.007*		23/34	0.89	(0.37,2.12)	0.793		0.69	(0.24,1.96)	0.487	
	29/33	0.39	(0.15,0.98)	0.045*	0.021*	0.4	(0.13,1.22)	0.109	0.037	48/34	2.41	(0.94,6.22)	0.068	0.138	2.09	(0.67,6.52)	0.203	0.367
PCB138	42/33	1				1				44/34	1				1			
	36/34	0.69	(0.34,1.38)	0.293		0.53	(0.22,1.25)	0.148		24/34	0.58	(0.29,1.13)	0.11		0.38	(0.17,0.87)	0.022	
	30/32	0.55	(0.24,1.27)	0.159		0.55	(0.2,1.54)	0.253		24/34	0.55	(0.25,1.19)	0.13		0.4	(0.16,1.02)	0.056	
	25/33	0.41	(0.17,1.03)	0.057	0.052	0.25	(0.08,0.81)	0.021*	0.033*	45/34	1.19	(0.54,2.63)	0.67	0.848	0.9	(0.33,2.47)	0.845	0.54
PCB153	42/34	1				1				47/34	1				1			
	38/32	0.73	(0.32,1.64)	0.444		0.59	(0.21,1.68)	0.324		19/34	0.39	(0.17,0.86)	0.02		0.4	(0.17,0.97)	0.041	
	30/33	0.5	(0.21,1.19)	0.115		0.52	(0.18,1.55)	0.243		28/34	0.61	(0.28,1.3)	0.198		0.55	(0.23,1.31)	0.175	
	23/33	0.35	(0.13,0.92)	0.034*	0.022*	0.18	(0.05,0.68)	0.011*	0.014*	43/34	0.89	(0.4,1.98)	0.774	1.00	0.68	(0.26,1.83)	0.45	0.537
PCB156	43/33	1				1				40/34	1				1			
	39/33	0.65	(0.31,1.36)	0.257		0.68	(0.27,1.76)	0.431		27/34	0.72	(0.34,1.51)	0.388		0.94	(0.41,2.18)	0.889	
	25/34	0.37	(0.15,0.9)	0.029*		0.34	(0.11,1.01)	0.053		26/34	0.69	(0.31,1.55)	0.369		0.8	(0.3,2.12)	0.652	
	26/32	0.42	(0.17,1.02)	0.054	0.032*	0.31	(0.1,1)	0.050*	0.021*	44/34	1.15	(0.48,2.75)	0.746	0.627	1.22	(0.42,3.52)	0.717	0.704
PCB170	39/34	1				1				41/34	1				1			
	38/33	0.86	(0.41,1.79)	0.679		0.8	(0.31,2.01)	0.629		28/34	0.71	(0.34,1.46)	0.347		0.87	(0.39,1.96)	0.737	
	27/33	0.56	(0.23,1.33)	0.185		0.55	(0.19,1.61)	0.275		28/34	0.61	(0.25,1.49)	0.283		0.68	(0.23,1.96)	0.473	
	29/32	0.64	(0.27,1.54)	0.321	0.217	0.38	(0.12,1.17)	0.092	0.066	40/34	0.92	(0.4,2.14)	0.854	1.00	0.94	(0.33,2.68)	0.906	0.905
PCB180	34/33	1				1				38/34	1				1			
	44/33	1.21	(0.57,2.55)	0.616		1.28	(0.47,3.44)	0.632		37/34	0.93	(0.44,1.96)	0.857		1.15	(0.48,2.77)	0.75	
	30/33	0.74	(0.31,1.72)	0.479		0.73	(0.25,2.15)	0.572		20/34	0.42	(0.17,1.06)	0.065		0.32	(0.1,1.03)	0.056	
	25/33	0.63	(0.26,1.53)	0.312	0.15	0.48	(0.16,1.49)	0.205	0.079	42/34	1.10	(0.46,2.66)	0.829	0.889	0.84	(0.25,2.81)	0.779	0.534
HCB	54/34	1				1				32/33	1				1			
	33/33	0.25	(0.09,0.69)	0.007*		0.25	(0.08,0.77)	0.016*		31/34	1.06	(0.48,2.3)	0.892		1.17	(0.47,2.93)	0.733	
	16/33	0.09	(0.03,0.3)	<0.001*		0.04	(0.01,0.21)	<0.001*		42/34	1.48	(0.6,3.67)	0.398		1.49	(0.48,4.63)	0.488	
	30/32	0.16	(0.05,0.54)	0.003	0.002*	0.07	(0.01,0.35)	0.001*	0.001*	32/34	1.14	(0.38,3.48)	0.812	0.647	1.26	(0.34,4.67)	0.731	0.688
DDE	44/33	1				1				37/34	1				1			
	37/33	0.64	(0.29,1.41)	0.267		0.46	(0.16,1.26)	0.13		33/34	0.82	(0.37,1.83)	0.626		0.53	(0.19,1.45)	0.216	
	25/34	0.37	(0.15,0.9)	0.028*		0.21	(0.07,0.65)	0.007*		35/34	0.83	(0.36,1.93)	0.672		0.4	(0.13,1.22)	0.107	
	27/32	0.39	(0.15,1.02)	0.054	0.031*	0.16	(0.04,0.6)	0.006*	0.003*	32/34	0.74	(0.28,1.96)	0.54	0.591	0.41	(0.11,1.46)	0.167	0.143

OR – conditional logistic regression accounting for matching factors

OR^(adj)^ – conditional logistic regression additionally adjusting for BMI, height, educational level, vegetables, dairy, protein, total fat, alcohol

Matched case-control pairs with incomplete information on confounders were excluded from analyses; *Significant at the 95% confidence level

Information on histological subtype was available for 225 of 270 cases (83%) and the remaining 45 cases were classified as ‘B-cell NHL, not otherwise specified’. The most common subtype was Multiple Myeloma (MM), which accounted for 28% of cases. The associations between log transformed exposure levels and risk for the four largest subgroups are shown in Table 5. The significant inverse associations were limited to Diffuse Large B-cell Lymphoma (DLBCL). However, when a meta-analyses of each exposure by subtype was conducted, the I^2^ was 0% for all exposures except PCB170 (19.1%) and PCB180 (30.1%), and the *p* values were >0.1, indicating no significant heterogeneity between the subtypes.

**Table 5:** Association between log-transformed body burden of six PCB congeners, specified PCB functional groups, HCB and DDE, levels and NHL risk stratified by Subtype

Exposure	CLL (*n* = 42)						DLBCL (*n* = 45)					
	OR	95% CI	*p-value*	OR^(adj)^	95% CI	*p-value*	OR	95% CI	*p-value*	OR^(adj)^	95% CI	*p-value*
PCB118	0.70	(0.31,1.58)	0.398	5.14	(0.56,47.08)	0.147	0.74	(0.27,2.04)	0.561	0.39	(0.06,2.68)	0.335
PCB138	0.63	(0.26,1.54)	0.309	1.33	(0.15,11.91)	0.801	0.37	(0.10,1.32)	0.126	0.33	(0.04,2.47)	0.281
PCB153	0.50	(0.16,1.52)	0.221	0.70	(0.06,7.77)	0.772	0.32	(0.08,1.26)	0.103	0.16	(0.01,1.84)	0.140
PCB156	0.57	(0.16,2.10)	0.402	1.47	(0.11,19.75)	0.773	0.25	(0.06,0.96)	0.044^*^	0.01	(0,0.53)	0.023^*^
PCB170	0.52	(0.16,1.66)	0.267	0.34	(0.03,3.61)	0.372	0.24	(0.06,1.02)	0.053	0.01	(0,0.47)	0.021^*^
PCB180	0.49	(0.14,1.77)	0.278	0.27	(0.02,4.15)	0.351	0.23	(0.05,0.97)	0.045^*^	0.01	(0,0.50)	0.020^*^
HCB	0.42	(0.14,1.26)	0.121	1.12	(0.17,7.40)	0.905	0.90	(0.34,2.39)	0.834	0.41	(0.08,2.00)	0.271
DDE	0.83	(0.44,1.58)	0.578	1.99	(0.52,7.59)	0.313	0.88	(0.43,1.84)	0.743	1.17	(0.35,3.97)	0.796
Exposure	FL (*n* = 39)						MM (*n* = 76)					
	OR	95% CI	*p-value*	OR^b(adj)^	95% CI	*p-value*	OR	95% CI	*p-value*	OR^b(adj)^	95% CI	*p-value*
PCB118	0.95	(0.41,2.21)	0.904	1.04	(0.34,3.13)	0.947	1.06	(0.55,2.07)	0.856	0.62	(0.23,1.63)	0.330
PCB138	0.91	(0.31,2.67)	0.865	1.17	(0.29,4.77)	0.822	0.74	(0.32,1.70)	0.474	0.45	(0.15,1.32)	0.145
PCB153	0.94	(0.29,3.04)	0.918	1.20	(0.27,5.31)	0.814	0.73	(0.29,1.85)	0.512	0.41	(0.12,1.42)	0.159
PCB156	0.99	(0.32,3.01)	0.979	1.12	(0.24,5.36)	0.885	0.77	(0.31,1.88)	0.561	0.47	(0.14,1.62)	0.234
PCB170	1.39	(0.43,4.51)	0.580	1.65	(0.33,8.24)	0.540	0.77	(0.30,2.00)	0.588	0.42	(0.11,1.66)	0.216
PCB180	1.43	(0.44,4.63)	0.553	1.52	(0.31,7.41)	0.605	0.90	(0.35,2.31)	0.834	0.56	(0.15,2.10)	0.392
HCB	0.82	(0.36,1.86)	0.633	0.83	(0.25,2.70)	0.752	0.70	(0.30,1.61)	0.394	0.31	(0.08,1.19)	0.089
DDE	0.77	(0.41,1.48)	0.437	0.93	(0.34,2.55)	0.885	0.66	(0.37,1.15)	0.142	0.52	(0.25,1.10)	0.086

Results for the four largest subtype groups were included

OR – conditional logistic regression accounting for matching factors

OR^(adj)^ – conditional logistic regression additionally adjusting for BMI, height, educational level, vegetables, dairy, protein, total fat, alcohol

^*^Significant at the 95% confidence level; Matched case-control pairs with incomplete information on potential confounders were excluded from the adjusted analyses

Additional stratified analyses were performed to identify confounders or effect modifiers that may explain the unexpected inverse associations. Neither age at recruitment (and therefore at blood draw; Additional file 1: Table S3) nor time to diagnosis (Additional file 1: Table S4) appeared to be modifying the findings. Results from the meta-analysis indicated that the percentage of heterogeneity not due to chance (I^2^) was 0% for all exposures, and all *p*-values were non-significant. When subjects were stratified by BMI (Additional file 1: Table S5) there was some indication that risk estimates tended to decrease with BMI category, with the smallest ORs generally noted in obese participants. Effect modification by BMI may also in part explain the observed sex discrepancy, 69.6% of males were overweight or obese compared with only 50.2% of females (*p* < 0.0001). However, there was no significant heterogeneity between BMI classes for any of the exposures (*P* > 0.05) and no conclusions can be drawn based on these analyses alone. The ORs were greater than one for PCBs 118, 153, 170 and 180 in individuals whose exposure levels were above the 90th percentile. This could be interpreted to suggest that very high levels may increase risk, however these associations were not significant, (Table 6).

**Table 6:** Association between exposure and risk in ‘highly exposed’ individuals high exposure is defined as exposure levels above the 90^th^ of the control exposure distribution, the odds of disease are compared with those in the lowest quartile of exposure

Exposure	90th percentile control exposure distribution	*n* cases (%) above 90^th^ percentile	*n* controls(%) above 90^th^ percentile	OR	95% CI	*p-*value	OR^(adj)^	95% CI	*p-*value
PCB118	316.2	35(13.0)	28(10.4)	1.37	(0.76,2.45)	0.294	1.25	(0.65,2.41)	0.500
PCB138	1112.4	25(9.3)	29(10.7)	0.83	(0.45,1.52)	0.550	0.81	(0.40,1.64)	0.562
PCB153	1965.7	29(10.7)	29(10.7)	1.01	(0.56,1.81)	0.987	1.15	(0.58,2.26)	0.693
PCB156	176.4	23(8.5)	28(10.4)	0.79	(0.43,1.45)	0.447	0.89	(0.43,1.81)	0.739
PCB170	626.6	29(10.7)	29(10.7)	1.00	(0.57,1.78)	0.989	1.07	(0.55,2.06)	0.843
PCB180	1323.3	29(10.7)	28(10.4)	1.05	(0.58,1.88)	0.872	1.11	(0.57,2.14)	0.763
HCB	872.8	25(9.3)	29(10.7)	0.80	(0.41,1.55)	0.506	0.84	(0.42,1.68)	0.624
DDE	9787.7	27(10)	28(10.4)	0.95	(0.50,1.80)	0.878	1.01	(0.52,1.97)	0.978

OR – conditional logistic regression accounting for matching factors

OR^(adj)^ – conditional logistic regression additionally adjusting for BMI, height, educational level, vegetables, dairy, protein, total fat, alcohol

Matched case-control pairs with incomplete information on potential confounders were excluded from the adjusted analyses

Groups of PCBs based on the properties of the congeners [18] were also analysed together; dioxin-like congeners (PCB#s 118, 156), non-dioxin like congeners (PCB#s138, 153, 170, 180) and immunotoxic congeners (PCB#s118, 138, 156, 170), and the sum total of all PCBs. However, these were driven by the constituent PCBs and significant inverse relationships were noted for the non-dioxin like and immunotoxic groups (results not shown). Categorising subjects into quartiles based on the distribution of exposure levels in the total population and exposure levels in cases only, did not change the findings (results not shown).

## Discussion

Exposure to PCBs and other persistent organic pollutants has been hypothesised to be partly responsible for the increase in NHL observed in the last 30 years [8, 19], which is larger than can be explained on the basis of changes to the classification procedures [19] or on known risk factors alone [20]. However the literature is inconclusive [21, 22]. In this study a prospective design was used to explore the risk of NHL associated with internal dose of six PCB congeners, HCB and DDE, under the hypothesis that an elevated body burden may increase future risk of this malignancy. We report geographical and sex-specific differences in results. In this population there was evidence of a possible increase in risk with exposure to certain PCB congeners in females, and a number of unexpected inverse associations in males. Our results additionally suggest possible differences in risk by subtype but these were based on small numbers. Stratified analyses revealed no evidence of confounding or effect modification by age at diagnosis or by time between blood draw and disease diagnosis. Those in the very highest levels of exposure showed a non-significantly increased risk for a number of PCBs.

PCBs, a mixture of synthetic chlorinated hydrocarbons that are the products and incidental by-products of multiple industrial and agricultural processes, have recently been upgraded to Class 1 carcinogens by the International Agency for Research on Cancer [23]. Although once widely used, concern about their health effects and toxicity has led to PCBs being banned in most countries [24]. However, these compounds are exceptionally stable, chemically inert and highly soluble in lipids [25]. Consequently they have extensively polluted the environment and are still found in all environmental media, including air, water, and soil which has led to bioaccumulation in the food chain, and most people have a measurable amount of PCBs in their bodies [21].

The evidence for an association with NHL is based on the observed tumorogenic and carcinogenic properties of PCBs, and in particular the dioxin-like properties exhibited by the coplanar congeners [26, 27]. The dioxin-like congeners share a biological mechanism, based on binding to the Ah-receptor [28], with 2,3,7,8-TCDD a compound which has previously been associated with an increased risk of lymphoid malignancies [29]. The association with NHL is further supported by the known immunosupressive and inflammatory properties of PCBs [30], and the temporal relationship between the incidence of NHL and the worldwide production of PCBs.

Although PCBs have been widely explored in relation to NHL, the evidence has been contradictory. While many studies report a positive association with the sum of PCB body burden, PCB mixtures (including dioxin-like, non-dioxin like and immunotoxic) and individual congeners [26, 31–36], an equally large number have found no association [37–43]. Many of the positive findings come from case-control studies which are subject to the problems of reverse causation and other bias, or from investigations of high levels of accidental exposure [36, 44]. Even among highly exposed occupational cohorts the results have not been consistent [22]. Others have utilised proxies, such as house dust, which may not accurately reflect actual exposure [35]. Of the studies most directly comparable with this study, those utilising prediagnostic exposure measurements in the general population, two [34, 45] reported an increased risk with total PCB exposure in both sexes, while a third [32] reported an increase in males. Two further studies [37, 38] reported no significant associations for any of the investigated congeners. Even systematic reviews have been unable to confirm or refute an association, and a causal relationship remains to be defined [21, 22, 25, 35, 45].

The literature is similarly inconsistent for DDE and for HCB, which showed some of the strongest inverse associations in this population. Three case control-studies have reported a positive association between NHL and DDE. One was based on adipose tissue concentrations [42] and two measured levels in blood [31, 33]. However, in a study of serum organochlorine levels in participants living near a municipal solid waste incinerator the relationship was borderline significant and the OR indicated only a marginal increase in risk [31]. Additionally a number of both case-control [26, 41] and cohort [32, 37, 38] studies have reported no association. Similarly, for those studies considering HCB; although one [33] observed a positive association with risk, two other studies report null findings [31, 37].

In this study of a general, non-occupationally exposed cohort, exposure was assessed using blood concentrations of eight POPs in samples taken from cancer-free individuals at recruitment to the cohort. This provides a point estimate of historical exposure free from recall bias, which can be common when individuals are surveyed on their long term exposure to environmental pollutants. Additionally, the measure is free from disease bias; pathological changes may affect the levels of certain pollutants and therefore distort the association, but in this study all participants were free of lymphoma at ascertainment. Global restrictions in the use of POPs has led to a reported decrease in human body burden over the last decade: for PCBs current exposure levels are estimated to be half those of 20–30 years ago [46], making it difficult to compare between populations and across periods. However, the observed exposure levels for the POPs were within the range of those reported in studies of similar populations [47]. All POPs, with the exception of PCB170, were at higher levels in the Italian cohort.

In general, inverse associations were noted for males, while females tended to show positive, although non-significant associations. Gender-specific differences were also noted in the studies by Bertrand and Laden [32, 38], however conversely to our findings they report a positive association in males but find no evidence of a relationship in females. Other studies of PCBs have observed no gender differences [34], however analysis of similar organochlorines suggest that sex-specific differences in the uptake, metabolism and elimination of compounds may result in variation in risk effects [37, 48–52] Breast feeding and parity are also likely to play a role [46].

The heterogeneous nature of NHL and the differing composition of subtypes explored in other studies may, in part, explain the differing findings between this study and previous work [42]. A number of the studies did not stratify their results by subtype [8, 9, 31, 34, 37, 40, 42, 45]. Among those that did the strongest associations tended to be noted for DLBCL [26, 32, 33], with weaker and in some cases (non-significantly) inverse associations reported for FL [26] and SLL/CLL [32, 38] in some studies. Even where specific subtypes have been studied the results have been inconsistent between studies. For example, in contrast to much of the literature FL has been reported to be strongly associated with PCBs in at least one study [33], while within a single study differing directions of effect were observed for DLBCL among different geographical populations [41]. Findings are further complicated by reports that subtype-specific associations may also differ by genotype [28] and by t(14:18) translocation status [42]. It should be noted that none of the existing studies reported MM as a separate subgroup, as due to its pathophysiological differences MM is often considered a distinct entity from other B-cell NHLs. However under the most commonly used classifications scheme today MM is classed as a B-cell NHL (hierarchical group 4) and therefore in this study was considered with the other B-cell subtypes. In the present study the inverse associations appeared to be driven by cases of DLBCL, however we report no significant evidence of subtype heterogeneity. The differing composition of the PCB congeners included in different studies may also account for some of the observed discrepancy between studies, particularly where a risk effect for the sum of PCBs has been reported. It is also noted that exposure to PCBs and how they are metabolised may differ in an occupational setting as opposed to in the general population [8].

In order to try and further disentangle our findings, a number of additional analyses were performed. To ensure that pre-diagnostic subclinical changes in cases who were diagnosed shortly after blood draw were not modifying the body burden of exogenous exposures [53], stratification by the time to diagnosis was performed. However there was no evidence of an effect, and similarly age at diagnosis did not appear to be modifying the results. There was, however, some weak evidence of an effect of BMI, which may be worthy of further investigation. Risk estimates tended to decrease with BMI category, with the smallest ORs generally noted in obese participants. This may be of interest as organochlorines are stored in adipose tissue and therefore PCB levels in the blood have previously been reported to be inversely associated with weight gain, and overweight and obese individuals have been observed to have lower circulating levels [54]; this may mask an association if POPs are most metabolically active on a tissue level. If BMI does act as a confounder this could explain the high number of inverse associations noted in this population which contained a high proportion (60%) of overweight and obese individuals. It would also explain why inverse associations were overrepresented in males, who had a significantly higher proportion of overweight and obese participants. Additional analyses would be required to determine whether BMI was driving the observed gender effects or vice versa. However, as noted in the results there was no significant heterogeneity between BMI classes.

In this study, lipids were not adjusted for as it has been observed that direct standardisation can introduce bias [55], and unadjusted volume-based concentrations of POPs have been found to correlate well with lipid based concentrations [15]. Furthermore, the possibility of ‘reverse causation’ and disease progression bias [53], which are the main reasons to adjust for lipids, (in order to take physiological pre-cancerous changes to normal metabolic process and lipid mobilisation into account), have been considered in these analyses. However, the findings for BMI suggest that lipid profiles of individuals may be important, and we cannot rule out that the lack of lipid-adjustment may be influencing our results.

The main strength of this study is its prospective design which protects against selection bias and it was determined that the case and control populations were similar with respect to baseline factors and additional confounders. This design also allows for the determination of a temporal and causal relationship between exposure and NHL, lacking in the majority of studies which tend to measure post-diagnostic or post-treatment levels. NHL or its treatment may affect metabolism and blood concentrations of POPs [32, 54], and chemotherapy has been observed to decrease PCB levels in the body by up to 30% [21]. The measures of exposure assessment were free from recall and disease bias, and were conducted using validated methodologies. The use of blood measurements has been shown to provide a reliable estimate of POP body burden due to their long half-lives [38].

There were several limitations to this study. The high correlation between exposures, particularly the PCBs, restricted the ability to determine individual effects [56]. When multiple POPs were investigated in combination no additional associations were revealed. It is possible that there were further unmeasured confounders affecting the results, for example other organochlorines or dioxins [45] which may bioaccumulate in parallel with the exposures of interest. Furthermore variables relating to the immune system, such as EBV antigens which have been shown to interact with organochlorines in NHL risk [39] can also not be accounted for. The estimate of body burden is based on a single one spot exposure measurement that is not necessarily representative of lifetime exposure. Finally, the generalizability of these findings to other populations should be considered, given the relative overrepresentation of females, the excess of MM cases and the relatively high proportion of overweight participants. In particular, these findings should not be extrapolated into an occupational setting.

These limitations may in part explain the unexpected nature of a number of the findings. However, there is some precedent in the literature. A study in Finland has previously suggested that dioxins may act on the risk of soft-tissue sarcoma through a J-shaped dose response curve, and there in some support from animal models for such a relationship [57]. The results of the ‘high exposure’ analysis could considered to be supportive of a J-shaped curve, however again these results were non-significant, and the results from the quartile analysis do not support this hypothesis within the confines of the exposure levels in this study population.

## Conclusion

In conclusion, we report no evidence that a higher body burden of PCBs, DDE or HCB increased the risk of subsequent NHL diagnosis. Significantly inverse associations were noted for males with a number of the investigated POPs. Despite extensive analyses we were unable to explain these associations. We additionally note that the associated *p*-values would not be robust for multiple testing, and that for the highest levels of exposure the relationship was null. This study adds to the existing body of literature and the results do not support a role for PCBs, DDE and HCB as risk factors for NHL.

## Additional file

Additional file 1: Tables S1–S5 with additional results can be found in the Additional file documents; RKelly_Additional_files.docx. (DOCX 72 kb)

## Abbreviations

BDE-47: 2,2',4,4'-Tetrabromodiphenyl ether
BMI: Body mass index
CLL/SLL: Chronic lymphocytic leukemia/ small lymphocytic lymphoma
DDE: Dichlorodiphenyldichloroethylene
DDT: Dichlorodiphenyltrichloroethane
DLBCL: Diffuse large B-cell lymphoma
EPIC: European Prospective Investigation into Cancer
FL: Follicular lymphoma
GC: Gas chromatography
HCB: Hexachlorobenzene
HCH: hexachlorocyclohexane
LOQ: Limit of quantification
MM: Multiple myeloma
MS: Mass spectrometry
NHL: Non-Hodgkin lymphoma
NSHDS: Northern Sweden Health and Disease Study
PCB: Polychlorinated biphenyl
POP: Persistent organic pollutant
SEER: Surveillance, epidemiology, and end results program
