# Supplementary material for: Prediagnostic plasma concentrations of organochlorines and risk of B-cell non-Hodgkin lymphoma in envirogenomarkers: a nested case-control study
Source: Environ Health. 2017 Feb 16;16:9. doi: 10.1186/s12940-017-0214-8 (PMC5312563; doi:10.1186/s12940-017-0214-8)
Supplement: Additional file 1: Tables S1–S5 — with additional results can be found in the Additional file documents; RKelly_Additional_files.docx. (DOCX 72 kb) [file 12940_2017_214_MOESM1_ESM.docx]

## ADDITIONAL FILES

**Prediagnostic serum concentrations of PCBs and organochlorines and risk of B-cell Non-Hodgkin Lymphoma in EnviroGenoMarkers: A nested case-control study**

**Authors:**

Rachel S. Kelly^1,2^ , Hannu Kiviranta ^3^, Ingvar A. Bergdahl^4^ , Domenico Palli^5^, Ann-Sofie Johansson^6^, Maria Botsivali^7^, Paolo Vineis^2,8^, Roel Vermeulen^9^, Soterios A. Kyrtopoulos^7^, Marc Chadeau-Hyam^2^  ,

*on behalf of the EnviroGenoMarkers project consortium*

### Additional Table S1: Pearson correlation coefficients (r^2^) between log transformed exposure concentrations of six PCB congeners, HCB and DDE

|  | **PCB118** | **PCB138** | **PCB153** | **PCB156** | **PCB170** | **PCB180** | **HCB** | **DDE** |
| --- | --- | --- | --- | --- | --- | --- | --- | --- |
| **PCB118** | 1.000 |  |  |  |  |  |  |  |
| **PCB138** | *0.775* | 1.000 |  |  |  |  |  |  |
| **PCB153** | *0.768* | *0.968* | 1.000 |  |  |  |  |  |
| **PCB156** | 0.654 | *0.794* | *0.880* | 1.000 |  |  |  |  |
| **PCB170** | 0.595 | *0.826* | *0.909* | *0.957* | 1.000 |  |  |  |
| **PCB180** | 0.635 | *0.810* | *0.903* | *0.928* | *0.972* | 1.000 |  |  |
| **HCB** | 0.688 | 0.454 | 0.473 | 0.420 | 0.357 | 0.445 | 1.000 |  |
| **DDE** | 0.733 | 0.674 | 0.634 | 0.389 | 0.386 | 0.484 | 0.732 | 1.000 |

*All correlation coefficients are statistically significant (p<0.001)*

*r2>0.7 are highlighted in italics to indicate high correlation*

### Additional Table S2: Association between NHL and quartiles of exposure to persistent **organic pollutants and metals stratified by cohort**

| **POP by quartile** | |  | **NSHDS** | | | | | | | | | |  | **EPIC-Italy** | | | | | | | | | |
| --- | --- | --- | --- | --- | --- | --- | --- | --- | --- | --- | --- | --- | --- | --- | --- | --- | --- | --- | --- | --- | --- | --- | --- |
|  |  |  | **Ca/Co** | **OR** | **95% CI** | **P-value** | ***p for trend*** |  | **OR^(adj)^** | **95% CI** | **P-value** | ***p for trend*** |  | **Ca/Co** | **OR** | **95% CI** | **P-value** | ***p for trend*** |  | **OR^(adj)^** | **95% CI** | **P-value** | ***p for trend*** |
|  |  |  |  |  |  |  |  |  |  |  |  |  |  |  |  |  |  |  |  |  |  |  |  |
| **PCB118** | 1 |  | 68/54 | **1** |  |  |  |  | **1** |  |  |  |  | 29/20 | **1** |  |  |  |  | **1** |  |  |  |
|  | 2 |  | 53/47 | **0.84** | (0.48,1.48) | 0.551 |  |  | **1.14** | (0.58,2.23) | 0.701 |  |  | 16/22 | **0.48** | (0.19,1.23) | 0.126 |  |  | **0.61** | (0.21,1.8) | 0.372 |  |
|  | 3 |  | 32/47 | **0.45** | (0.23,0.88) | 0.019* |  |  | **0.36** | (0.16,0.82) | 0.014* |  |  | 14/21 | **0.46** | (0.17,1.27) | 0.133 |  |  | **0.5** | (0.17,1.5) | 0.216 |  |
|  | 4 |  | 33/37 | **0.61** | (0.32,1.17) | 0.14 | *0.047** |  | **0.4** | (0.16,0.99) | 0.047* | *0.012** |  | 25/20 | **1.08** | (0.37,3.16) | 0.895 | *0.672* |  | **1.12** | (0.31,4.01) | 0.864 | *0.754* |
| **PCB138** | 1 |  | 53/46 | **1** |  |  |  |  | **1** |  |  |  |  | 34/21 | **1** |  |  |  |  | **1** |  |  |  |
|  | 2 |  | 48/47 | **0.85** | (0.48,1.52) | 0.579 |  |  | **0.67** | (0.33,1.36) | 0.268 |  |  | 11/20 | **0.28** | (0.09,0.81) | 0.020* |  |  | **0.17** | (0.05,0.63) | 0.008* |  |
|  | 3 |  | 41/46 | **0.68** | (0.35,1.33) | 0.262 |  |  | **0.48** | (0.21,1.1) | 0.082 |  |  | 18/22 | **0.38** | (0.13,1.14) | 0.084 |  |  | **0.27** | (0.07,0.98) | 0.046* |  |
|  | 4 |  | 44/46 | **0.75** | (0.38,1.5) | 0.418 | *0.344* |  | **0.47** | (0.2,1.15) | 0.097 | *0.064* |  | 21/20 | **0.44** | (0.13,1.47) | 0.181 | *0.198* |  | **0.32** | (0.07,1.47) | 0.143 | *0.136* |
| **PCB153** | 1 |  | 54/46 | **1** |  |  |  |  | **1** |  |  |  |  | 29/21 | **1** |  |  |  |  | **1** |  |  |  |
|  | 2 |  | 46/47 | **0.75** | (0.39,1.42) | 0.376 |  |  | **0.76** | (0.36,1.61) | 0.48 |  |  | 19/21 | **0.57** | (0.22,1.48) | 0.245 |  |  | **0.46** | (0.15,1.38) | 0.166 |  |
|  | 3 |  | 44/47 | **0.66** | (0.32,1.35) | 0.251 |  |  | **0.62** | (0.25,1.51) | 0.294 |  |  | 13/20 | **0.31** | (0.09,1.08) | 0.066 |  |  | **0.22** | (0.05,0.95) | 0.043* |  |
|  | 4 |  | 42/45 | **0.66** | (0.31,1.39) | 0.276 | *0.282* |  | **0.39** | (0.15,1) | 0.05 | *0.048** |  | 23/21 | **0.5** | (0.15,1.71) | 0.272 | *0.33* |  | **0.46** | (0.11,1.93) | 0.285 | *0.361* |
| **PCB156** | 1 |  | 46/46 | **1** |  |  |  |  | **1** |  |  |  |  | 28/21 | **1** |  |  |  |  | **1** |  |  |  |
|  | 2 |  | 59/47 | **1.16** | (0.59,2.28) | 0.666 |  |  | **1.46** | (0.67,3.17) | 0.343 |  |  | 21/20 | **0.67** | (0.23,1.9) | 0.448 |  |  | **0.46** | (0.13,1.61) | 0.227 |  |
|  | 3 |  | 32/47 | **0.62** | (0.28,1.35) | 0.23 |  |  | **0.56** | (0.21,1.47) | 0.242 |  |  | 11/22 | **0.3** | (0.09,0.99) | 0.049* |  |  | **0.16** | (0.03,0.72) | 0.017* |  |
|  | 4 |  | 49/45 | **1** | (0.47,2.14) | 0.995 | *0.638* |  | **0.75** | (0.3,1.89) | 0.544 | *0.241* |  | 24/20 | **0.61** | (0.16,2.26) | 0.458 | *0.377* |  | **0.42** | (0.08,2.11) | 0.291 | *0.294* |
| **PCB170** | 1 |  | 45/47 | **1** |  |  |  |  | **1** |  |  |  |  | 27/21 | **1** |  |  |  |  | **1** |  |  |  |
|  | 2 |  | 61/46 | **1.31** | (0.7,2.44) | 0.403 |  |  | **1.22** | (0.59,2.51) | 0.595 |  |  | 19/20 | **0.6** | (0.21,1.7) | 0.336 |  |  | **0.4** | (0.12,1.36) | 0.141 |  |
|  | 3 |  | 34/46 | **0.73** | (0.36,1.51) | 0.403 |  |  | **0.5** | (0.2,1.3) | 0.155 |  |  | 17/21 | **0.45** | (0.13,1.49) | 0.19 |  |  | **0.34** | (0.08,1.38) | 0.13 |  |
|  | 4 |  | 46/46 | **0.96** | (0.47,1.96) | 0.92 | *0.529* |  | **0.64** | (0.25,1.6) | 0.339 | *0.16* |  | 21/21 | **0.51** | (0.14,1.82) | 0.299 | *0.368* |  | **0.41** | (0.09,1.83) | 0.241 | *0.382* |
| **PCB180** | 1 |  | 45/47 | **1** |  |  |  |  | **1** |  |  |  |  | 26/21 | **1** |  |  |  |  | **1** |  |  |  |
|  | 2 |  | 59/46 | **1.34** | (0.71,2.56) | 0.367 |  |  | **1.41** | (0.65,3.05) | 0.382 |  |  | 19/20 | **0.74** | (0.3,1.81) | 0.504 |  |  | **0.48** | (0.16,1.43) | 0.187 |  |
|  | 3 |  | 35/46 | **0.76** | (0.37,1.55) | 0.444 |  |  | **0.48** | (0.19,1.26) | 0.139 |  |  | 20/22 | **0.6** | (0.19,1.87) | 0.378 |  |  | **0.44** | (0.11,1.68) | 0.23 |  |
|  | 4 |  | 47/46 | **1** | (0.5,2.01) | 0.996 | *0.651* |  | **0.63** | (0.24,1.64) | 0.342 | *0.168* |  | 19/20 | **0.62** | (0.2,1.96) | 0.414 | *0.447* |  | **0.45** | (0.11,1.89) | 0.274 | *0.336* |

**Additional Table S2** ***continued***

| **POP by quartile** | |  | **NSHDS** | | | | | | | | | |  | **EPIC-Italy** | | | | | | | | | |
| --- | --- | --- | --- | --- | --- | --- | --- | --- | --- | --- | --- | --- | --- | --- | --- | --- | --- | --- | --- | --- | --- | --- | --- |
|  |  |  | **Ca/Co** | **OR** | **95% CI** | **P-value** | ***p for trend*** |  | **OR^(adj)^** | **95% CI** | **P-value** | ***p for trend*** |  | **Ca/Co** | **OR** | **95% CI** | **P-value** | ***p for trend*** |  | **OR^(adj)^** | **95% CI** | **P-value** | ***p for trend*** |
|  |  |  |  |  |  |  |  |  |  |  |  |  |  |  |  |  |  |  |  |  |  |  |  |
| **HCB** | 1 |  | 53/46 | **1** |  |  |  |  | **1** |  |  |  |  | 27/21 | **1** |  |  |  |  | **1** |  |  |  |
|  | 2 |  | 49/46 | **0.8** | (0.42,1.53) | 0.496 |  |  | **0.9** | (0.42,1.9) | 0.774 |  |  | 18/20 | **0.64** | (0.27,1.51) | 0.305 |  |  | **0.68** | (0.27,1.73) | 0.419 |  |
|  | 3 |  | 36/46 | **0.53** | (0.24,1.2) | 0.129 |  |  | **0.51** | (0.18,1.4) | 0.19 |  |  | 20/22 | **0.61** | (0.23,1.64) | 0.329 |  |  | **0.69** | (0.24,2) | 0.489 |  |
|  | 4 |  | 48/46 | **0.71** | (0.31,1.6) | 0.408 | *0.424* |  | **0.5** | (0.18,1.4) | 0.189 | *0.156* |  | 19/20 | **0.6** | (0.2,1.85) | 0.377 | *0.368* |  | **0.59** | (0.17,2.07) | 0.414 | *0.411* |
| **DDE** | 1 |  | 68/47 | **1** |  |  |  |  | **1** |  |  |  |  | 20/21 | **1** |  |  |  |  | **1** |  |  |  |
|  | 2 |  | 41/46 | **0.5** | (0.26,0.95) | 0.035* |  |  | **0.39** | (0.18,0.86) | 0.020* |  |  | 19/20 | **1.01** | (0.38,2.66) | 0.984 |  |  | **1.18** | (0.42,3.34) | 0.753 |  |
|  | 3 |  | 39/46 | **0.45** | (0.23,0.89) | 0.022* |  |  | **0.33** | (0.14,0.78) | 0.011* |  |  | 22/22 | **1.05** | (0.41,2.7) | 0.915 |  |  | **1.25** | (0.44,3.53) | 0.672 |  |
|  | 4 |  | 38/46 | **0.46** | (0.24,0.89) | 0.022* | *0.026** |  | **0.19** | (0.07,0.48) | 0.001* | *<0.001** |  | 23/20 | **1.34** | (0.46,3.86) | 0.589 | *0.607* |  | **1.44** | (0.43,4.79) | 0.551 | *0.555* |

### Additional Table S3; Association between log-transformed body burden of six PCB congeners, specified PCB functional groups, HCB and DDE levels and NHL risk stratified by age group at recruitment

| **Exposure** |  | **30-44 years (Cases n=40, Controls n=40)** | | |  | **45-59 years (Cases n=169, Controls n=158)** | | |  | **60-75 years (Cases n=61, Controls n=72)** | | |
| --- | --- | --- | --- | --- | --- | --- | --- | --- | --- | --- | --- | --- |
|  |  | **OR** | **95% CI** | ***p-value*** |  | **OR** | **95% CI** | ***p-value*** |  | **OR** | **95% CI** | ***p-value*** |
| **PCB118** |  | **0.39** | (0.11,1.35) | *0.136* |  | **0.89** | (0.58,1.37) | *0.597* |  | **1.48** | (0.69,3.17) | *0.308* |
| **PCB138** |  | **0.39** | (0.10,1.52) | *0.176* |  | **0.80** | (0.50,1.28) | *0.347* |  | **0.98** | (0.39,2.48) | *0.968* |
| **PCB153** |  | **0.33** | (0.07,1.51) | *0.152* |  | **0.76** | (0.45,1.30) | *0.323* |  | **0.83** | (0.31,2.22) | *0.710* |
| **PCB156** |  | **0.15** | (0.02,0.97) | *0.047** |  | **0.81** | (0.44,1.52) | *0.516* |  | **0.85** | (0.31,2.33) | *0.747* |
| **PCB170** |  | **0.31** | (0.07,1.42) | *0.130* |  | **0.85** | (0.47,1.55) | *0.596* |  | **0.79** | (0.26,2.45) | *0.686* |
| **PCB180** |  | **0.36** | (0.08,1.64) | *0.187* |  | **0.93** | (0.51,1.70) | *0.812* |  | **0.79** | (0.25,2.48) | *0.688* |
| **HCB** |  | **0.47** | (0.13,1.74) | *0.257* |  | **0.76** | (0.46,1.24) | *0.267* |  | **0.62** | (0.21,1.82) | *0.389* |
| **DDE** |  | **0.80** | (0.28,2.30) | *0.685* |  | **0.85** | (0.63,1.14) | *0.270* |  | **0.86** | (0.50,1.48) | *0.579* |
|  |  |  |  |  |  |  |  |  |  |  |  |  |
|  |  | **OR^(adj)^** | **95% CI** | ***p-value*** |  | **OR^(adj)^** | **95% CI** | ***p-value*** |  | **OR^(adj)^** | **95% CI** | ***p-value*** |
| **PCB118** |  | **0.36** | (0.04,3.04) | *0.345* |  | **0.72** | (0.43,1.23) | *0.235* |  | **1.10** | (0.4,3.01) | *0.858* |
| **PCB138** |  | **0.43** | (0.03,5.67) | *0.521* |  | **0.59** | (0.31,1.13) | *0.110* |  | **0.70** | (0.19,2.54) | *0.584* |
| **PCB153** |  | **0.44** | (0.02,9.11) | *0.593* |  | **0.55** | (0.27,1.15) | *0.115* |  | **0.71** | (0.2,2.53) | *0.596* |
| **PCB156** |  | **0.21** | (0.01,4.22) | *0.310* |  | **0.70** | (0.32,1.55) | *0.382* |  | **0.90** | (0.25,3.29) | *0.874* |
| **PCB170** |  | **0.63** | (0.05,8.89) | *0.736* |  | **0.67** | (0.31,1.44) | *0.308* |  | **0.82** | (0.18,3.77) | *0.804* |
| **PCB180** |  | **0.97** | (0.04,22.46) | *0.983* |  | **0.76** | (0.35,1.64) | *0.483* |  | **0.87** | (0.19,3.99) | *0.860* |
| **HCB** |  | **0.45** | (0.04,4.71) | *0.506* |  | **0.69** | (0.39,1.22) | *0.198* |  | **0.30** | (0.06,1.54) | *0.150* |
| **DDE** |  | **1.16** | (0.13,10.58) | *0.898* |  | **0.65** | (0.44,0.98) | *0.038** |  | **0.53** | (0.24,1.16) | *0.114* |

*OR – conditional logistic regression accounting for matching factors OR^(adj)^ – conditional logistic regression additionally adjusting for BMI, height, educational level, vegetables, dairy, protein, total fat, alcohol*

**Significant at the 95% confidence level*

### Additional Table S4; Association between log-transformed body burden of six PCB congeners, specified PCB functional groups, HCB, and DDE, levels and NHL risk stratified by time to diagnosis

| **Exposure** |  | **ttd< 5 years (n=108)** | | |  | **ttd> 5 years (n=149)** | | |  | **ttd< 5 years (n=108)** | | |  | **ttd> 5 years (n=149)** | | |
| --- | --- | --- | --- | --- | --- | --- | --- | --- | --- | --- | --- | --- | --- | --- | --- | --- |
|  |  | **OR** | **95% CI** | ***p-value*** |  | **OR** | **95% CI** | ***p-value*** |  | **OR^(adj)^** | **95% CI** | ***p-value*** |  | OR^(adj)^ | **95% CI** | ***p-value*** |
| **PCB118** |  | **0.82** | (0.52,1.27) | *0.368* |  | **0.95** | (0.64,1.41) | *0.808* |  | **0.75** | (0.47,1.22) | *0.253* |  | 0.88 | (0.55,1.39) | *0.576* |
| **PCB138** |  | **0.63** | (0.39,1.01) | *0.056* |  | **0.73** | (0.46,1.14) | *0.166* |  | **0.57** | (0.34,0.95) | *0.032** |  | 0.63 | (0.36,1.1) | *0.105* |
| **PCB153** |  | **0.59** | (0.34,1.02) | *0.058* |  | **0.67** | (0.39,1.13) | *0.129* |  | **0.54** | (0.3,0.98) | *0.042** |  | 0.58 | (0.31,1.08) | *0.084* |
| **PCB156** |  | **0.67** | (0.36,1.27) | *0.22* |  | **0.7** | (0.4,1.22) | *0.204* |  | **0.68** | (0.34,1.33) | *0.26* |  | 0.65 | (0.35,1.23) | *0.186* |
| **PCB170** |  | **0.61** | (0.33,1.15) | *0.125* |  | **0.71** | (0.4,1.28) | *0.258* |  | **0.59** | (0.3,1.16) | *0.129* |  | 0.62 | (0.32,1.22) | *0.169* |
| **PCB180** |  | **0.62** | (0.33,1.17) | *0.141* |  | **0.77** | (0.43,1.39) | *0.385* |  | **0.59** | (0.29,1.18) | *0.138* |  | 0.7 | (0.35,1.39) | *0.310* |
| **HCB** |  | **0.63** | (0.38,1.06) | *0.082* |  | **0.74** | (0.46,1.21) | *0.232* |  | **0.62** | (0.36,1.07) | *0.086* |  | 0.65 | (0.37,1.14) | *0.130* |
| **DDE** |  | **0.73** | (0.54,0.99) | *0.045** |  | **0.79** | (0.59,1.06) | *0.118* |  | **0.65** | (0.47,0.92) | *0.013** |  | 0.71 | (0.51,1.01) | *0.054* |

*OR – conditional logistic regression accounting for matching factors OR^(adj)^ – conditional logistic regression additionally adjusting for BMI, height, educational level, vegetables, dairy, protein, total fat, alcohol*

**Significant at the 95% confidence level*

*ttd-time to diagnosis*

### Additional Table S5; Association between log-transformed body burden of six PCB congeners, specified PCB functional groups, HCB and DDE, levels and NHL risk stratified by BMI category

| **Exposure** |  | **Normal (Cases n=105, Controls n=109)** | | |  | **Overweight (Cases n=121, Controls n=118)** | | |  | **Obese (Cases n=40, Controls n=39)** | | |
| --- | --- | --- | --- | --- | --- | --- | --- | --- | --- | --- | --- | --- |
|  |  | **OR** | **95% CI** | ***p-value*** |  | **OR** | **95% CI** | ***p-value*** |  | **OR** | **95% CI** | ***p-value*** |
| **PCB118** |  | **0.95** | (0.43,2.13) | *0.906* |  | **0.58** | (0.28,1.22) | *0.152* |  | **0.49** | (0.06,3.72) | *0.488* |
| **PCB138** |  | **0.77** | (0.36,1.64) | *0.497* |  | **0.16** | (0.05,0.6) | *0.006** |  | **0.21** | (0.02,2.87) | *0.242* |
| **PCB153** |  | **0.74** | (0.31,1.8) | *0.511* |  | **0.17** | (0.04,0.67) | *0.011** |  | **0.07** | (0,2.91) | *0.16* |
| **PCB156** |  | **0.78** | (0.29,2.1) | *0.621* |  | **0.35** | (0.1,1.16) | *0.085* |  | **0.07** | (0,3.1) | *0.17* |
| **PCB170** |  | **0.83** | (0.31,2.27) | *0.721* |  | **0.34** | (0.1,1.13) | *0.078* |  | **3.55x10^-3^** | (0,9.16) | *0.159* |
| **PCB180** |  | **0.83** | (0.29,2.34) | *0.721* |  | **0.31** | (0.09,1.04) | *0.058* |  | **2.24x10^-3^** | (0,8.67) | *0.148* |
| **HCB** |  | **1.23** | (0.45,3.36) | *0.688* |  | **0.35** | (0.12,1.03) | *0.056* |  | **2.46x10^-3^** | (0,6.06) | *0.132* |
| **DDE** |  | **0.83** | (0.45,1.5) | *0.529* |  | **0.28** | (0.12,0.66) | *0.003** |  | **0.42** | (0.06,3.23) | *0.407* |
|  |  |  |  |  |  |  |  |  |  |  |  |  |
|  |  | **OR^(adj)^** | **95% CI** | ***p-value*** |  | **OR^(adj)^** | **95% CI** | ***p-value*** |  | **OR^(adj)^** | **95% CI** | **p-value** |
| **PCB118** |  | **0.68** | (0.19,2.45) | *0.551* |  | **0.49** | (0.18,1.3) | *0.153* |  | **∆** |  |  |
| **PCB138** |  | **0.53** | (0.12,2.28) | *0.393* |  | **0.08** | (0.01,0.49) | *0.007** |  |  |  |  |
| **PCB153** |  | **0.62** | (0.12,3.25) | *0.572* |  | **0.12** | (0.02,0.68) | *0.016** |  |  |  |  |
| **PCB156** |  | **1.06** | (0.26,4.27) | *0.934* |  | **0.28** | (0.06,1.25) | *0.095* |  |  |  |  |
| **PCB170** |  | **1.44** | (0.26,7.94) | *0.678* |  | **0.22** | (0.04,1.12) | *0.069* |  |  |  |  |
| **PCB180** |  | **1.44** | (0.24,8.51) | *0.685* |  | **0.19** | (0.04,0.97) | *0.046** |  |  |  |  |
| **HCB** |  | **1.62** | (0.4,6.65) | *0.502* |  | **0.19** | (0.04,0.96) | *0.044** |  |  |  |  |
| **DDE** |  | **0.54** | (0.22,1.35) | *0.186* |  | **0.11** | (0.02,0.53) | *0.006** |  |  |  |  |

*OR – conditional logistic regression accounting for matching factors OR^(adj)^ – conditional logistic regression additionally adjusting for BMI, height, educational level, vegetables, dairy, protein, total fat, alcohol*

**Significant at the 95% confidence level*

**∆** *model would not converge*
